#  Low-temperature catalyst activator: mechanism of dense carbon nanotube forest growth studied using synchrotron radiation

**DOI:** 10.1107/S2052252514009907

**Published:** 2014-05-22

**Authors:** Akito Takashima, Yudai Izumi, Eiji Ikenaga, Takuo Ohkochi, Masato Kotsugi, Tomohiro Matsushita, Takayuki Muro, Akio Kawabata, Tomo Murakami, Mizuhisa Nihei, Naoki Yokoyama

**Affiliations:** a Japan Synchrotron Radiation Research Institute (JASRI), 1-1-1 Kouto, Sayo, Hyogo 679-5198, Japan; b Collaborative Research Team Green Nanoelectronics Center (GNC), National Institute of Advanced Industrial Science and Technology (AIST), 16-1 Onogawa, Tsukuba, Ibaraki 305-8569, Japan

**Keywords:** dense vertically aligned carbon nanotubes, thermal chemical vapor deposition, growth mechanism, synchrotron radiation, soft X-ray photoemission spectroscopy (SXPES), hard X-ray photoemission spectroscopy (HAXPES), photoemission electron microscope (PEEM), X-ray absorption spectroscopy (XAS)

## Abstract

The mechanism of the one-order-of-magnitude increase in the density of vertically aligned carbon nanotubes (CNTs) achieved by a recently developed thermal chemical vapor deposition process was studied using synchrotron radiation spectroscopic techniques. In the developed process, a Ti film is used as the underlayer for an Fe catalyst film. A characteristic point of this process is that C_2_H_2_ feeding for the catalyst starts at a low temperature of 450°C, whereas conventional feeding temperatures are ∼800°C. Photoemission spectroscopy using soft and hard X-rays revealed that the Ti underlayer reduced the initially oxidized Fe layer at 450°C. A photoemission intensity analysis also suggested that the oxidized Ti layer at 450°C behaved as a support for nanoparticle formation of the reduced Fe, which is required for dense CNT growth. In fact, a CNT growth experiment, where the catalyst chemical state was monitored *in situ* by X-ray absorption spectroscopy, showed that the reduced Fe yielded a CNT forest at 450°C. Contrarily, an Fe layer without the Ti underlayer did not yield such a CNT forest at 450°C. Photoemission electron microscopy showed that catalyst annealing at the conventional feeding temperature of 800°C caused excess catalyst agglomeration, which should lead to sparse CNTs. In conclusion, in the developed growth process, the low-temperature catalyst activation by the Ti underlayer before the excess Fe agglomeration realised the CNT densification.

## Abstract 2

The mechanism of dense vertically aligned carbon nanotube growth achieved by a recently developed thermal chemical vapor deposition method was studied using synchrotron radiation spectroscopic techniques.

## 1. Introduction   

Carbon nanotubes (CNTs) have a wide range of potential applications including stiff composites, energy storage, sensors and electronics, owing to their outstanding mechanical, electrical and thermal properties (De Volder *et al.*, 2013 ▶). For example, the thermal conductivity of a multiwalled CNT was reported to be more than 3000 W m^−1^ K^−1^ at room temperature (RT) (Kim *et al.*, 2001 ▶), which makes CNTs a potential candidate as a next-generation heat dissipation material. Excess heat generation in devices has become one of the most critical problems to be conquered for further progress of electronics. CNTs are promising for thermal applications, such as thermal interface materials (TIMs) placed between heatsinks and CPU chips or thermal through-silicon vias (TTSVs) that dissipate heat from local hot spots in large-scale integration (LSI) devices. Since the high thermal transport of each CNT is obtained along the CNT axis, an aggregate of directionally arrayed CNTs, such as a CNT forest vertically grown on a substrate, is required to minimize the total conductivity of a TIM or a TTSV. There have been many experimental reports on the thermal conductivities of CNT forests (Yang *et al.*, 2004 ▶; Ivanov *et al.*, 2006 ▶; Shaikh *et al.*, 2007 ▶; Lin *et al.*, 2012 ▶). Most of them are, however, less than 50 W m^−1^ K^−1^ and inferior to solders that are currently often used as TIMs. Amin-Chalhoub *et al.* recently reported a value of 180 W m^−1^ K^−1^ (Amin-Chalhoub *et al.*, 2012 ▶). This value is comparable with the thermal conductivity of Si at RT (Glassbrenner & Slack, 1964 ▶), but still insufficient because the thermal conductivity of a TTSV should be much higher than that of the surrounding Si. As suggested by Lin *et al.* (2012 ▶), a major factor that determines the thermal conductivity is the packing density of CNTs. Technical improvements in densifying CNTs are desired.

Thermal chemical vapor deposition (TCVD) is a growth method for CNT forests (Robertson *et al.*, 2012 ▶). A typical process of TCVD is as follows. A film of a transition metal such as Fe deposited on a substrate is used as a catalyst. By annealing it under vacuum, typically up to ∼800°C, the catalyst film is transformed into catalytically activated nanoparticles. Then, a carbon feedstock such as C_2_H_2_ is dosed to the nanoparticles, forming CNTs. Usually, a film of an oxide such as Al_2_O_3_ is laid under the catalyst film as a support for the nanoparticle formation. Because the density of the CNTs is determined by that of the catalyst nanoparticles, particle densification leads to dense CNTs. Plasma pretreatment often works effectively to form dense nanoparticles (Choi *et al.*, 2003 ▶; Hofmann *et al.*, 2005 ▶; Wen *et al.*, 2005 ▶; Yamazaki *et al.*, 2010 ▶; Zhang *et al.*, 2013 ▶). Chemical activation of the catalyst is also imperative because CNTs do not grow on inactive particles. Hofmann *et al.* studied Fe catalysts by photoemission spectroscopy (PES) and concluded that the active state is metallic Fe (Hofmann *et al.*, 2009 ▶). Thus, in many cases, pretreatments of initially oxidized Fe with reductants such as H_2_ or NH_3_ are carried out (Choi *et al.*, 2003 ▶; Hofmann *et al.*, 2005 ▶; Wen *et al.*, 2005 ▶; Yamazaki *et al.*, 2010 ▶; Zhang *et al.*, 2013 ▶). Although all these pretreatments are often effective, they complicate the growth process, which is unfavorable for the integration of TCVD into industrial applications.

Recently, we developed an improved TCVD process that can densify CNTs without any pretreatments (Kawabata *et al.*, 2013 ▶). We use Fe and Ti films with thicknesses of 2 and 1 nm as the catalyst layer and underlayer, respectively, which are deposited on a Si substrate. Hereafter, we refer to this catalyst system as Fe/Ti. In the improved process, we start to feed C_2_H_2_ at 450°C and continue the feeding during the subsequent temperature elevation to 800°C. As a result, the CNT density obtained by this process was 20 times higher than that obtained by the conventional one where C_2_H_2_ was supplied at 800°C (Kawabata *et al.*, 2013 ▶). A high thermal conductivity of 260 W m^−1^ K^−1^ was also achieved for a grown CNT forest (Kawabata *et al.*, 2013 ▶). We named the improved process ‘STEP’ as an abbreviation of ‘slope control of temperature profile’ (Kawabata *et al.*, 2013 ▶). The growth results suggest that, in the STEP process, catalyst activation and nanoparticle formation occurred at or near 450°C. However, this temperature seems unusually low to reduce oxidized Fe. The used Fe/Ti was exposed to air before TCVD. Maeda *et al.* studied the chemical states of an ∼1 nm-thick oxidized Fe film on a Si substrate using PES (Maeda *et al.*, 2004 ▶). The measured Fe 2*p* spectra were dominated by Fe oxides up to 525°C and finally a small metallic Fe peak appeared at 580°C (Maeda *et al.*, 2004 ▶), which is much higher than the feeding start temperature of 450°C in the STEP process. It is still unknown how the STEP process densified the CNTs, which should be understood for further densification.

In the present work, in order to address the mechanism of the CNT densification by the STEP process, we studied the temperature dependence of the morphological and chemical states of Fe/Ti using synchrotron radiation. We used photoemission electron microscopy (PEEM) to element-specifically observe the surface morphological change of the Fe catalyst (Swiech *et al.*, 1997 ▶). The chemical states were investigated using PES. For the Fe layer, we used soft X-ray PES (SXPES) with a probing depth comparable with the Fe layer thickness. To probe the buried Ti layer, hard X-ray photoemission spectroscopy (HAXPES) (Takata *et al.*, 2005 ▶) with an increased probing depth was also used. The results revealed important roles of the Ti layer for the reduction and deformation of the Fe layer at 450°C. Finally, to clarify whether the Fe catalyst of Fe/Ti is actually activated at 450°C, we performed CNT growth experiments at this temperature, where the Fe chemical states were monitored *in situ* by soft X-ray absorption spectroscopy (XAS). The results were dramatically different between the cases with and without the Ti layer, indicating the critical role of the Ti layer for the Fe catalyst activation. We also examined the carbon products *in situ* by C 1*s* XAS, which is sensitive to local structures of carbon materials (Comelli *et al.*, 1988 ▶).

## 2. Experimental   

**Figure 1:**
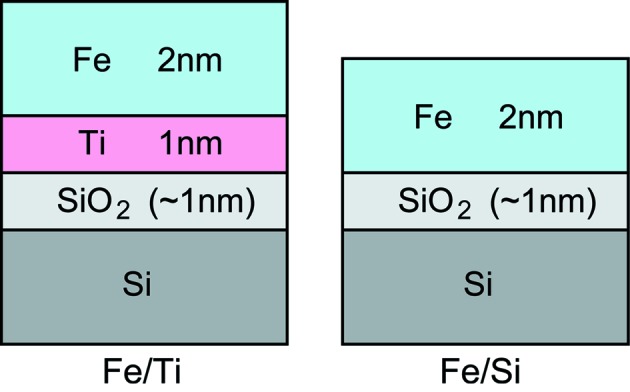
Schematic drawing of the samples.

The samples are schematically described in Fig. 1 ▶. For Fe/Ti, Fe and Ti layers with thicknesses of 2 and 1 nm, respectively, were deposited on a Si substrate by electron beam evaporation. For a comparative sample, a 2 nm-thick Fe layer was directly deposited on a Si substrate, which is hereafter denoted as Fe/Si. The Si substrates had natural surface SiO_2_ layers approximately 1 nm thick. The pressure during the evaporation was 5 × 10^−5^ Pa. The samples were exposed to air for a few days before the spectroscopic analyses.

All the spectroscopic experiments were performed at SPring-8. PEEM images on the Fe/Ti and Fe/Si surfaces were observed at BL17SU using a SPELEEM of Elmitec GmbH with a spatial resolution of ∼50 nm (Guo *et al.*, 2007 ▶). A photon energy (*h*ν) of 708 eV at the Fe 2*p*_3/2_ absorption edge was used to element-specifically observe the morphological change of the Fe catalyst (Swiech *et al.*, 1997 ▶). The base pressure was 8 × 10^−7^ Pa. SXPES measurements using an *h*ν of 1500 eV were performed for Fe/Ti and Fe/Si at BL27SU (Ohashi *et al.*, 2001 ▶). In the case of Fe 2*p* SXPES with this *h*ν, the probing depth is expected to be ∼1.4 nm (Tanuma *et al.*, 2011 ▶). A PHOIBOS 150 analyzer of SPECS GmbH was used. The total energy resolution was 310 meV. The beam spot size on the sample surface was ∼10 µm × 200 µm. Ti 2*p* HAXPES measurements with an *h*ν of 7940 eV were carried out for Fe/Ti at BL47XU (Ikenaga *et al.*, 2013 ▶). In this case, the probing depth reaches ∼8 nm (Tanuma *et al.*, 2011 ▶), which can probe the full thickness of the buried Ti layer. An R4000 analyzer of VG Scienta AB was used. The total energy resolution was 250 meV. The base pressures were 3.0 × 10^−6^ and 2.0 × 10^−6^ Pa for SXPES and HAXPES, respectively.

The CNT growth experiments were performed at BL27SU. Before supplying C_2_H_2_, Fe 2*p* XAS spectra of Fe/Ti and Fe/Si were measured at RT and 450°C under a vacuum of ∼1 × 10^−5^ Pa, to observe the Fe chemical states. The partial electron yield method was used for the XAS measurements, where electrons with kinetic energies higher than 500 eV were detected. In this case, the probing depth of XAS is expected to be ∼1−1.2 nm (Tanuma *et al.*, 2011 ▶), which is close to that of SXPES. After the Fe 2*p* XAS measurements, C_2_H_2_ gas diluted with Ar was introduced into the chamber for 7.5 min, keeping the pressure at 1 kPa and the temperature at 450°C. C 1*s* XAS spectra were measured *in situ* for both samples kept at 450°C after evacuating the chamber again. The pressure during the measurements was 2 × 10^−5^ Pa. We used linearly polarized light and set the X-ray **E** vector at 35° with respect to the substrate normal. Then, the samples were taken out from the chamber and observed using a scanning electron microscope (SEM).

## 3. Results and discussion   

**Figure 2:**
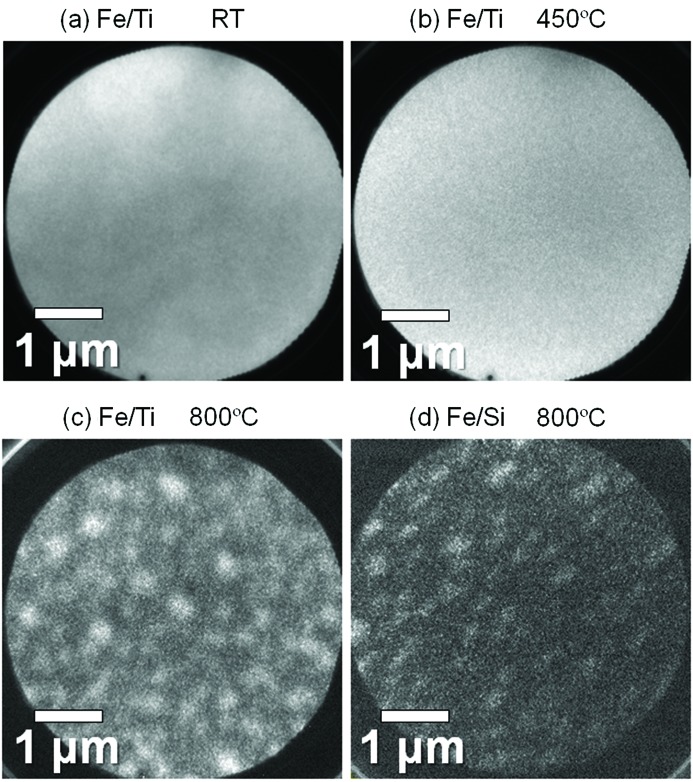
PEEM images on the Fe/Ti surface at (*a*) RT, (*b*) 450°C and (*c*) 800°C, observed at the Fe 2*p*_3/2_ absorption edge. (*d*) PEEM image on the Fe/Si surface at 800°C.

Figs. 2(*a*), 2(*b*) and 2(*c*) ▶ show the PEEM images on Fe/Ti observed using an *h*ν of 708 eV at RT, 450°C and 800°C, respectively. Brighter areas indicate higher Fe concentration. The Fe layer at 450°C in Fig. 2(*b*) ▶ seems homogeneous and not much different from that at RT in Fig. 2(*a*) ▶. In contrast, the image at 800°C in Fig. 2(*c*) ▶ shows much lower surface occupation of Fe, where Fe forms islands with sizes of a few hundred nanometers. The low Fe occupation could be due to excess agglomeration or diffusion into the underlayer and/or substrate. It is considered that this low Fe occupation resulted in the low CNT density for the conventional process, where C_2_H_2_ was supplied at 800°C (Kawabata *et al.*, 2013 ▶). The image on Fe/Si observed at 800°C in Fig. 2(*d*) ▶ shows a similar behavior of island formation, but it shows a much lower Fe surface occupation. This implies that the Fe diffusion was relatively prevented in the case with the Ti layer. Although the image on Fe/Ti at 450°C in Fig. 2(*b*) ▶ seems homogeneous, there is a possibility that Fe nanoparticles with sizes of less than the spatial resolution of ∼50 nm were formed. We will return to this point later.

**Figure 3:**
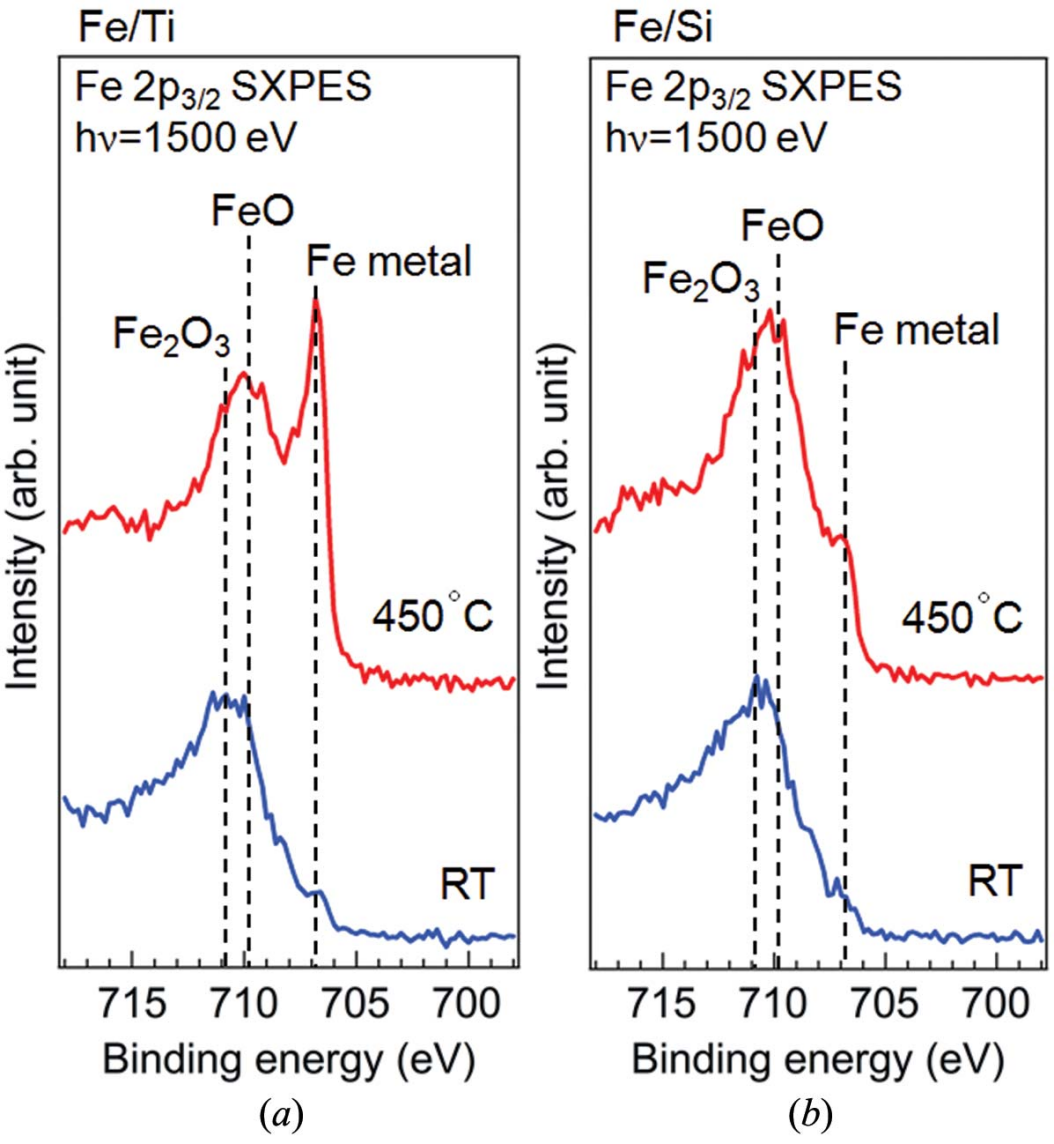
Fe 2*p*_3/2_ SXPES spectra of (*a*) Fe/Ti and (*b*) Fe/Si. Blue and red lines are the spectra measured at RT and 450°C, respectively.

Figs. 3(*a*) and 3(*b*) ▶ show the Fe 2*p*_3/2_ SXPES spectra of Fe/Ti and Fe/Si, respectively. The spectra at RT for both samples are similar and dominated by peaks at a binding energy (*E*_B_) of ∼710.8 eV, which is close to the reported *E*_B_ values of Fe^3+^ states of Fe_2_O_3_ (Brundle *et al.*, 1977 ▶; Graat & Somers, 1996 ▶; Fujii *et al.*, 1999 ▶; Gota *et al.*, 1999 ▶). In the Fe/Ti spectrum at 450°C in Fig. 3(*a*) ▶, a sharp peak of metallic Fe is prominently increased at 706.8 eV (Graat & Somers, 1996 ▶), and it almost dominates the spectrum. In addition, the oxide peak is shifted to 709.8 eV, indicating that the valency of iron is reduced to that of FeO (Brundle *et al.*, 1977 ▶; Graat & Somers, 1996 ▶; Gota *et al.*, 1999 ▶). In contrast, for Fe/Si at 450°C in Fig. 3(*b*) ▶, the increase of the Fe metal peak is much less prominent than in the case of Fe/Ti. Although some energy shift is also seen for the oxide peak of Fe/Si, the spectrum is still dominated by the oxide peak. This result for Fe/Si is consistent with the results reported by Maeda *et al.* (2004 ▶). The relatively enhanced Fe metal peak observed for Fe/Ti at 450°C suggests that the Ti layer has an important effect on the reduction of the Fe layer.

**Figure 4:**
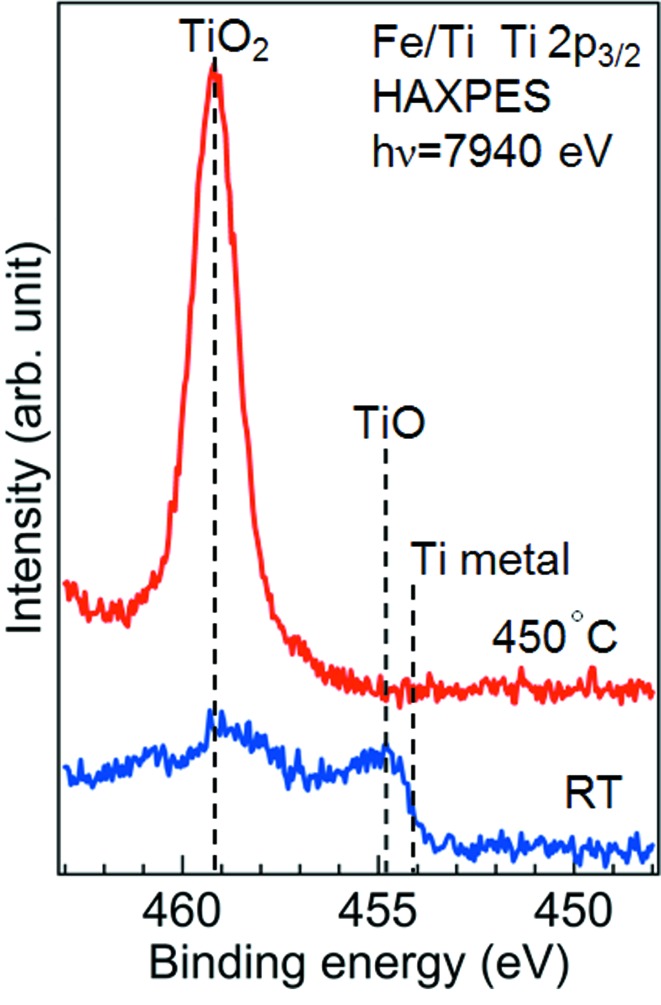
Ti 2*p*_3/2_ HAXPES spectra of Fe/Ti. Blue and red lines are the spectra at RT and 450°C, respectively.

Fig. 4 ▶ shows the Ti 2*p*_3/2_ HAXPES spectra of Fe/Ti at RT and 450°C. The spectrum at RT consists of two dominant peaks at 459.2 and 454.8 eV, which correspond to TiO_2_ and TiO, respectively (Mayer *et al.*, 1995 ▶; Bartkowski *et al.*, 1997 ▶). The intensity of the Ti metal component located at 454.1 eV (Mayer *et al.*, 1995 ▶) is very small. We used metallic Ti for the evaporant but the Ti layer proved to be partially oxidized. In the spectrum at 450°C, the TiO peak vanishes and a sharp TiO_2_ peak solely dominates the spectrum. This result, combined with the fact that the Fe metal peak at 450°C was relatively enhanced for Fe/Ti as observed in Fig. 3 ▶, clearly indicates that the Ti layer absorbed the oxygen of the Fe layer and prompted its reduction. This is convincing from a thermodynamic point of view, because the heat of formation per oxygen atom for TiO_2_ is −4.9 eV, which is approximately two times lower than the value of −2.8 eV for Fe_2_O_3_ (Lide, 1999 ▶). The initial partial oxidation of the Ti layer may be caused by oxygen diffusion through the Fe layer during the air exposure. Some oxidation could also have occurred during the evaporation because of the somewhat low vacuum of 5 × 10^−5^ Pa.

**Figure 5:**
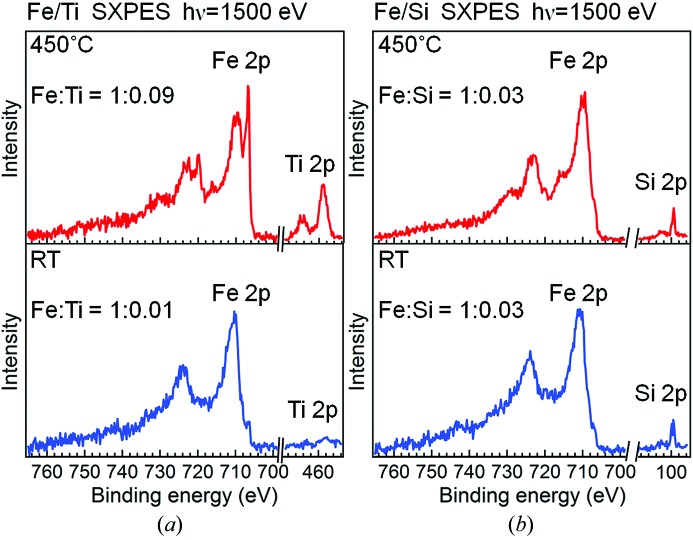
(*a*) Fe 2*p* and Ti 2*p* SXPES spectra of Fe/Ti at RT and 450°C. (*b*) Fe 2*p* and Si 2*p* SXPES spectra of Fe/Si at the corresponding temperatures. Shirley-type backgrounds are subtracted from all the spectra. The indicated ratios are those of integrated spectral intensities.

In addition to the catalyst reduction, nanoparticle formation is also indispensable for CNT growth, as already mentioned. We now discuss this point using the spectra of SXPES which is relatively surface-sensitive compared with HAXPES. Generally, when an overlayer is transformed into nanoparticles and part of the underlayer surface becomes uncovered, the intensity of surface-sensitive PES from the underlayer should be increased. In Fig. 5(*a*) ▶ the background-subtracted SXPES spectra of Fe/Ti in the Fe 2*p* and Ti 2*p* core regions at RT and 450°C are shown. The spectra at each temperature were extracted from a wide-range spectrum so that their intensities could be compared directly. Fig. 5(*b*) ▶ compares the Fe 2*p* and Si 2*p* SXPES spectra of Fe/Si in a similar way. In Fig. 5(*a*) ▶ the photoemission intensity in the Ti 2*p* region at RT is very weak as compared with that in the Fe 2*p* region, because the Ti layer is covered with the 2 nm-thick Fe layer. The integrated intensity ratio of Fe to Ti is 1:0.01. At 450°C, however, the Ti 2*p* peak intensity is significantly increased and the ratio of Fe to Ti becomes 1:0.09. In contrast, in Fig. 5(*b*) ▶ the intensity ratio of Fe to Si at 450°C is 1:0.03 and is not different from the ratio of 1:0.03 at RT. From the phase diagram of the Fe–Ti binary system (Okamoto, 2000 ▶), it is expected that Fe–Ti alloying does not occur by the temperature increase from RT to 450°C. Therefore, these results suggest that island-like deformation of the Fe layer occurred for Fe/Ti and part of the Ti layer surface was uncovered at 450°C. The homogeneous PEEM image at 450°C in Fig. 2(*b*) ▶ also implies that the island sizes are smaller than the spatial resolution of ∼50 nm and are thus suitable for CNT growth. In general, the surface free energies of metals are much higher than those of oxides. Thus, metals on oxide substrates tend to dewet the substrates. In fact, Fe deposited on TiO_2_(110) shows island growth up to a deposition thickness of a few monolayers (Pan & Madey, 1993 ▶). Therefore, the Fe layer of Fe/Ti with its dominant metallic portion could have dewetted on the completely oxidized Ti at 450°C.

**Figure 6:**
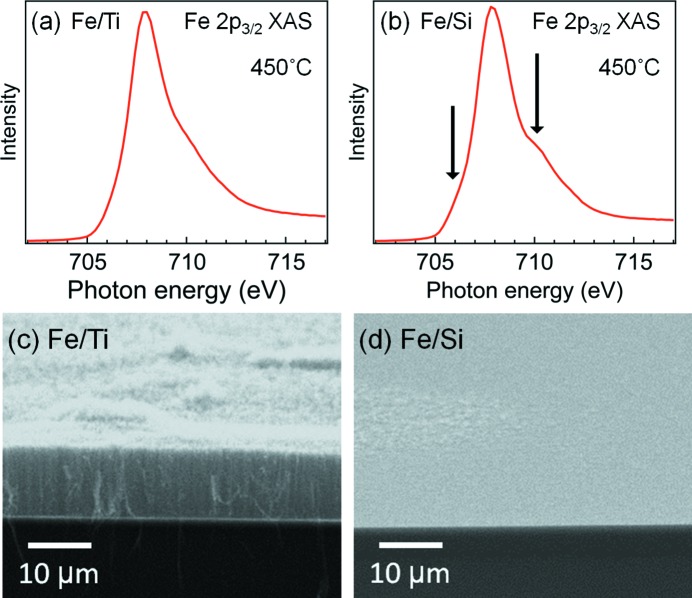
Fe 2*p*_3/2_ XAS spectra measured for (*a*) Fe/Ti and (*b*) Fe/Si at 450°C. (*c*) and (*d*) are the SEM images of Fe/Ti and Fe/Si, respectively, after the C_2_H_2_ exposure.

Finally, to examine the capability of CNT growth for Fe/Ti and Fe/Si at 450°C, we carried out growth experiments. Fig. 6(*a*) and 6(*b*) ▶ show the XAS spectra measured in the Fe 2*p*_3/2_ region for Fe/Ti and Fe/Si, respectively, at 450°C before supplying C_2_H_2_. The spectral shapes of both samples at RT, not shown here, were similar to those of Fe_2_O_3_ (Regan *et al.*, 2001 ▶; Kang *et al.*, 2008 ▶). The spectral shape of Fe/Si at 450°C in Fig. 6(*b*) ▶ reflects that of FeO (Regan *et al.*, 2001 ▶; Kang *et al.*, 2008 ▶), which is characterized by the shoulder structures indicated in the figure. In the Fe/Ti spectrum in Fig. 6(*a*) ▶, the shoulder structures are smoothed out and the spectral shape is closer to that of Fe metal (Regan *et al.*, 2001 ▶; Kang *et al.*, 2008 ▶). Therefore, for both Fe/Ti and Fe/Si, the temperature dependence of the Fe chemical states indicated by the XAS spectra are consistent with that indicated by the SXPES spectra in Fig. 3 ▶. Then, we introduced C_2_H_2_ to the samples kept at 450°C. Figs. 6(*c*) and 6(*d*) ▶ depict the SEM images for Fe/Ti and Fe/Si, respectively, after the C_2_H_2_ exposure. A CNT forest with CNT lengths of ∼10 µm was grown on Fe/Ti. However, Fe/Si did not yield such a CNT forest. These results clearly show that the Fe catalyst of Fe/Ti was actually activated at 450°C and the Ti layer had a critical role for this low-temperature activation. In other words, the Fe reduction prompted by the partially oxidized Ti made it possible to form a CNT forest at 450°C. It is also suggested that nanoparticle deformation could have occurred for the Fe layer at 450°C, which is usually required for CNT growth, supporting our deduction from the SXPES analysis.

**Figure 7:**
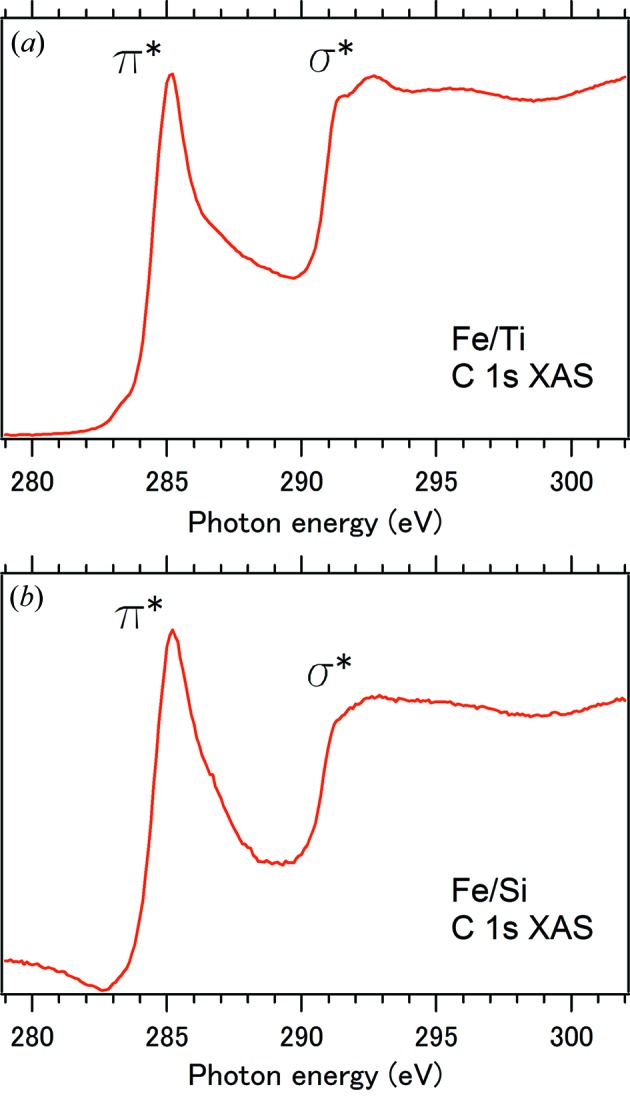
C 1*s* XAS spectra measured *in situ* for (*a*) Fe/Ti and (*b*) Fe/Si, after the C_2_H_2_ exposure.

Fig. 7 ▶ shows the C 1*s* XAS spectra measured *in situ* for Fe/Ti and Fe/Si after the C_2_H_2_ exposure. The spectrum of Fe/Ti in Fig. 7(*a*) ▶ shows a π* exciton peak at 285.1 eV and σ* absorption structures above 290 eV (Comelli *et al.*, 1988 ▶; Brühwiler *et al.*, 1995 ▶; Ahuja *et al.*, 1996 ▶). A σ* exciton structure is also clearly observed as a shoulder at 291.4 eV (Brühwiler *et al.*, 1995 ▶; Ahuja *et al.*, 1996 ▶). These features are characteristic to carbon materials with *sp* ^2^ bonding networks forming hexagonal carbon lattices such as highly oriented pyrolytic graphite (Comelli *et al.*, 1988 ▶; Brühwiler *et al.*, 1995 ▶; Ahuja *et al.*, 1996 ▶). Such features are also seen in the reported spectra of CVD-grown CNTs (Hemraj-Benny *et al.*, 2006 ▶; Li *et al.*, 2007 ▶). It is noteworthy that C—H bonding structures, which often appear at 287.5 eV (Comelli *et al.*, 1988 ▶), are not observed in Fig. 7(*a*) ▶. The spectrum of Fe/Si in Fig. 7(*b*) ▶ also shows π* and σ* absorption structures, indicating that some graphitic materials were also produced on Fe/Si. However, the π* peak width of Fe/Si is wider than that of Fe/Ti. Furthermore, the σ* exciton structure is smoothed out in the spectrum of Fe/Si. Such broadening trends are usually observed for amorphous-like graphitic carbon (Comelli *et al.*, 1988 ▶; Díaz *et al.*, 2001 ▶). This implies that the Fe layer of Fe/Si was not chemically and/or morphologically activated enough to produce well ordered hexagonal carbon lattices. These XAS results give the verification of better crystallized CNT growth for Fe/Ti in addition to the morphological SEM results in Fig. 6 ▶. The difference of the relative π* and σ* intensities between Fe/Ti and Fe/Si is considered to be due to the dependence on the liner polarization of the incident X-rays as previously observed for CNT forests (Hemraj-Benny *et al.*, 2006 ▶; Li *et al.*, 2007 ▶).

There have been several reports on low-temperature CVD CNT growth. We should mention the difference between them and our low-temperature growth presented here. Cantoro *et al.* reported that NH_3_ or H_2_ exposure facilitated the nanostructuring of Fe films, which enabled CNT growth at temperatures as low as 350°C (Cantoro *et al.*, 2006 ▶). Nessim *et al.* used preheating of the gas mixture of C_2_H_4_ and H_2_ at 770°C to grow crystalline CNTs at a substrate temperature of 500°C (Nessim *et al.*, 2009 ▶). These techniques require catalyst treatments with gaseous reductants, whereas our low-temperature catalyst activation of Fe/Ti requires only annealing in vacuum. Our results also show that CNT forests can be produced without preheating of C_2_H_2_ even at 450°C, although the used feedstock gas is different from that used by Nessim *et al.* Chen *et al.* also reported on CNT growth at substrate temperatures below 350°C (Chen *et al.*, 2011 ▶). However, they used infrared radiation heating from the front of a catalyst film to increase the temperature of the catalyst much higher than that of the substrate water-cooled from behind. Thus, their approach is different from our one in which the substrate is simply put on a heated plate.

**Figure 8:**
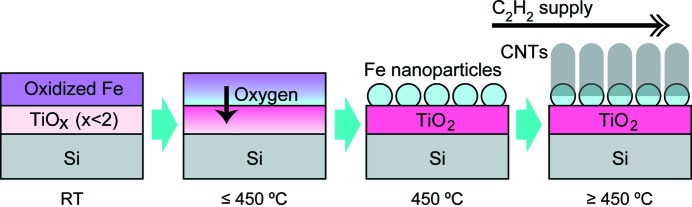
Mechanism of the dense CNT growth by the STEP process. The SiO_2_ layer is not drawn for simplicity.

We now summarize the chemical and morphological changes of Fe/Ti in the STEP process in Fig. 8 ▶. At RT, the Fe layer is almost fully oxidized and the Ti layer is partially oxidized. As the temperature is increased to 450°C, the oxygen in the Fe layer is absorbed by the Ti layer, as directly observed by SXPES and HAXPES. Then, the reduced Fe layer transforms into dense nanoparticles being supported by the fully oxidized Ti layer, although this is still our deduction from the SXPES, PEEM and CNT growth results. When C_2_H_2_ is supplied, dense seed CNTs are formed on the Fe nanoparticles. This high CNT density is expected to be maintained even during the subsequent temperature elevation for increasing the growth rate, because the root of each CNT holds a nanoparticle, preventing particle sintering. It is also expected that the TiO_2_ layer behaves as a diffusion barrier at the elevated temperatures as we observed in Fig. 2 ▶ that Fe/Ti showed a higher Fe surface occupation than Fe/Si at 800°C. We consider that this is the mechanism of the CNT densification achieved by the STEP process.

## 4. Conclusions   

We investigated the mechanism of the CNT densification achieved by the STEP process using the synchrotron radiation spectroscopic techniques of PEEM, SXPES, HAXPES and XAS. It was revealed that the Ti underlayer activated the Fe catalyst and made it possible to grow vertically aligned CNTs at 450°C, while conventional CNT growth is performed at ∼800°C. We conclude that this low-temperature catalyst activation by the Ti layer realised the CNT densification in the STEP process. This densification technique can use *ex situ* deposited and initially oxidized catalyst without any catalyst pretreatments. This is very advantageous for industrial applications from the viewpoint of production costs. We hope that our findings in this study will facilitate further optimization of the growth process conditions and the development of high-performance heat dissipation materials using CNTs.
